# NEDD1 Promotes the Development of Lung Adenocarcinoma and Can be Used as a Prognostic Marker

**DOI:** 10.7150/jca.98238

**Published:** 2024-08-13

**Authors:** Zhen Yang, Rongkun Huang, Yun Yang, Jing Cong, Xia Yang, Quan Zhou, Ruoyu Guo, Ying Ma, Xiangqian Gong, Fei Jiao, Youjie Li, Shugang Zhu, Lijun Kong, Jinxia Hu

**Affiliations:** 1 Key Laboratory of Tumor Molecular Biology in Binzhou Medical University, Department of Biochemistry and Molecular Biology, Binzhou Medical University, Yantai, Shandong 264003, China.; 2 Institute of Healthcare and Industry, Sichuan Tourism College, Chengdu, Sichuan 610000, China.; 3 Jining Medical University, Jining, Shandong 272067, China.; 4 Yantai Center for Disease Control and Prevention, Yantai, Shandong 264003, China.; 5 Department of Gastrointestinal Surgery, Yuhuangding Hospital, Yantai, Shandong 265499, China.; 6 Department of Cardiology, Zhaoyuan people's Hospital, Yantai, Shandong 265499, China.; 7 Department of Burn and Plastic Surgery, Yantai Affiliated Hospital of Binzhou Medical University, Shandong, China.

**Keywords:** NEDD1, lung cancer, migration, proliferation

## Abstract

**Objective***:* To explore the roles of Neural precursor cell expressed developmentally down-regulated 1(NEDD1) in lung cancer tumorigenesis and the relationship between NEDD1 expression and clinicopathology of patients with lung adenocarcinoma (LUAD).

**Methods**: Expression of NEDD1 or other proteins in tissues and cell lines were determined with immunohistochemistry or western blot, the data of patients with LUAD in The Cancer Genome Atlas (TCGA) datasets and LUAD tissue array were collected and analyzed, the effects of NEDD1 on proliferation, migration, cell cycle progression and apoptosis of cancer cells were detected with colony formation assay, transwell assay and Flow cytometry (FCM) analysis respectively. the impact of NEDD1 knockdown on DNA damage was analyzed using Immunofluorescence staining of H2AX and comet assay. Furthermore, the effect of NEDD1 on cancer cell proliferation *in vivo* was investigated in nude mice.

**Results***:* NEDD1 was upregulated in lung tissues and the NEDD1 immune score was an independent prognostic factor. Overexpression of NEDD1 promoted epithelial-mesenchymal transition, accelerated cell cycle progression, and enhanced the proliferation and migration of A549 and H1299 cells, while knockdown of NEDD1 resulted in the opposite phenotype and leaded to DNA damage. In addition, NEDD1 improved cell tumorigenicity* in vivo*.

**Conclusion***:* These findings suggest that NEDD1 plays important roles in lung cancer development and may therefore be a potential prognostic marker and promising therapeutic target for lung cancer therapy.

## Introduction

Although significant progress has been made in the early screening and treatment of lung cancer, lung cancer mortality rates remain higher than all other cancers. Lung adenocarcinoma (LUAD) is a significant classification of lung cancer. In China, the 5-year survival rate of lung cancer patients is only about 20% due to the high burden and the high proportion of late-stage lung cancer 1. Therefore, identifying the driving genes of lung cancer, developing effective therapeutic targets, and exploring new detection technologies are important goals of lung cancer research 2.

DNA and the centrosomes should be duplicated once per cell cycle, and therefore centrosome formation is a crucial step in mitosis. The centrosome is the locus of microtubule nucleation; failure of this process leads to monopolar or multipolar spindle formation, resulting in chromosome instability and cell apoptosis 3. A few proteins involved in centrosome formation have been reported as tumor promoters, and they may play essential roles in cancer development and progression due to their abnormal expression in cancer cells 4.

Neural precursor cell expressed developmentally down-regulated 1 (NEDD1), a highly conserved gene in plants and animals located at chromosome 12q22, codes a 55 kD protein containing a WD40 domain at the N-terminus and a coiled-coil domain at the C-terminus 5, 6. It has been reported that proteins containing the WD40 domain act as scaffolds and interact with other molecules to form huge complexes via the WD domain 7. NEDD1 is a member of the γ-tubulin ring complex (γ-TuRC) and interacts with γ-tubulin through the C-terminus, anchoring it to the centrosome, which is a critical step in microtubule nucleation and spindle assembly 8, including mitosis. These biological functions of NEDD1 are finely regulated by the phosphorylation of several kinases such as Polo-like kinase 1 (PLK1) 9, NimA related protein kinase9 (Nek9) 10, cyclin-dependent kinase 1 (Cdk1) 11, and Aurora A 12, 13. These kinases are important regulators of carcinogenesis. Standard NEDD1 expression is reportedly critical for accurate chromosome segregation in mammalian oocytes 14, mouse embryonic development 7, 15, and cell division in Arabidopsis thaliana 16. Furthermore, NEDD1 has been considered a tumor suppressor gene due to its chromosomal location 6, as ectopic expression of NEDD1 caused growth suppression in different cultured cells 5. Moreover, even though knockdown of NEDD1 using RNAi was reported to prolong the survival of scirrhous gastric cancer model mice 17, the role of NEDD1 in lung cancer development is not clear.

In the current study, we investigated the expression of NEDD1 in lung cancer, analyzed the relationship between clinicopathological characteristics and NEDD1 expression at mRNA and protein levels, and explored the role of NEDD1 in lung carcinogenesis.

## Materials and Methods

### Cell Culture

293T cells were purchased from the American Type Culture Collection (ATCC). A549 and H1299 cells were obtained from the National Collection and Authenticated Cell Culture of China, both cell lines have been authenticated by short tandem repeat profiling. All cell lines were cultured in Dulbecco's modified Eagle's medium or RPMI-1640 medium supplemented with 10% fetal bovine serum (all from Gibco, Thermo Fisher Scientific, Inc.) at 37 °C in a humidified atmosphere containing 5% CO2.

### RNAi and Transfection

RNA interference sequences of NEDD1 were: F-5'-GGGCAAAAGCAGACAUGUGTT-3' and R-5'-CACAUGUCUGCUUUUGCCCTT-3'. Transfection of expression plasmids or short interfering RNAs (siRNAs) into A549, H1299, and 293T cells was carried out using LipofectamineTM 3000 (Thermo Fisher Scientific, Waltham, MA, USA; L3000015) ac-cording to the manufacturer's recommendations. All siRNAs were purchased from GenePharma (Shanghai, China) and were transfected into cells at a final concentration of 50 nM.

### Lentivirus Production and Stable Overexpressing Cell Line Construction Establishment

One night before transfection, 293T cells were seeded in 10 cm plates. Lentivirus vector plasmid and lentivirus overexpression plasmid of NEDD1 were mixed with packing plasmids and transfection reagent (vigo) in DMEM medium, and then added into the medium of 293T cells for 6-8 h. The medium was then replaced with complete medium. The supernatants were collected at 24 h, 48 h, and 72 h and new medium was added every time. The viruses were then concentrated overnight. The lentivirus suspension was centrifuged at 5,000 rpm for 30 min at 4 °C. The pellet was collected and 100 µL PBS was added gently to resuspend the lentivirus vector virions to avoid air bubbles.

To establish stable cell line overexpressing NEDD1, A549 and H1299 cells were infected with lentivirus for 48 h, then, the cells were cultured in complete medium containing puromycin (2 µg/mL). After 72 hours, the overexpression of NEDD1 in A549 and H1299 cells was verified through western blot.

### Western Blot Analysis

Cells/tissues were collected and lysed with RIPA lysis buffer (Beyotime Institute of Biotechnology, Shanghai, China). The lysates were mixed with SDS protein sample buffer and denatured at 95 °C for 10 min. Proteins were separated by 10% SDS-PAGE and transferred onto polyvinylidene fluoride membranes (Sigma-Aldrich, USA). The membranes were incubated with 5% dried milk solution in TBST for 1 h and soaked in primary and secondary antibodies at 4 °C. All protein expression signals were detected using an automatic image analysis system (Tanon 5200 Multi, Shanghai, China) following electrochemiluminescence immune reactions. The primary antibodies and their working concentrations were as follows: anti-E-cadherin (1:1000; #9782, Cell Signaling, USA), anti-vimentin (1:1000; #9782, Cell Signaling, USA), anti-snail (1:1000; #9782, Cell signaling, USA), anti-NEDD1(1:1000, 13993-1-AP, Proteintech, Wuhan), anti-P-ATR (1:1000; #2853, Cell Signaling Technology, USA), anti-P-ATM (1:1000; #13050, Cell Signaling Technology, USA), anti-P-Chk1(1:1000; #2348 Cell Signaling Technology, USA), anti-P-Chk2(1:1000; #2197, Cell Signaling Technology, USA), anti-P-BRCA1 (1:1000; #9009, Cell Signaling Technology, USA), anti-P-H2A.X (1:1000; #9718, Cell signaling Technology, USA), anti-GAPDH (1:1000, BS65529, Bioworld Technology, USA).

### Cell Proliferation/Viability Assay

Cells transfected with siRNAs or virous were seeded into 96-well plates at a density of 5×10^3^ cells/well. After 48 h, 10 µL MTT (5 mg/mL, Sigma-Aldrich) was added to each well. The medium was removed 4 h later, 100 µL dimethyl sulfoxide (Sigma-Aldrich) was added, and the OD value (570 nm) was detected using a microplate reader (Multiskan FC, Thermo Fisher Scientific). Each experiment was performed in triplicate and repeated at least three times.

### Colony Formation Assays

Cells transfected with the indicated siRNAs were maintained in culture medium for 14 days at 37. Colonies containing more than 20 cells were counted. The colonies were fixed with 4% paraformaldehyde and stained with 0.1% crystal violet, and visible colonies were imaged and manually counted.

### Cell invasion Assays

Firstly, 600 µL medium with 20% FBS was added to the lower chamber, and 1×10^5^ cells in 100 µL serum-free medium were seeded into the upper chamber and incubated for 48 h. The non-migrated cells were removed with a cotton plug and the migrated cells fixed with 4% PFA, were stained with crystal violet. Finally, the migrated cells were observed under a microscope (Olympus, Tokyo, Japan). Images were acquired from five fields in each group.

### Apoptosis Assays

Cells were harvested and stained with Annexin V-FITC/PI (KeyGEN Biotech. Co., Ltd., Nanjing, China) according to the manufacturer's instructions and counted using flow cytometry (Beckman Coulter, Brea, CA, USA). Each experiment was performed in triplicate and repeated three times.

### Cell Cycle Analysis

Cells were collected and washed twice with pre-cold PBS and then fixed with cold 75% ethanol at -20 °C for more than 2 h. The fixed cells were resuspended in PBS containing RNaseA (50 U/mL) at 37 °C for 30 min, and then stained with propidium iodide (50 µg/mL) for 30 min at 4 °C. Finally, the cell cycle was detected by flow cytometry (BD Pharmingen, NJ, USA).

### Immunofluorescence Analysis

Cells growing on glass coverslips were fixed with 4% paraformaldehyde solution for 30 min and permeabilized with 0.1% NP-40 (Sigma, BioChemika). The slides were incubated with γH2AX antibody (C2036S-4, 1:100 in PBS, Beyotime, China) overnight at 4 °C and then blocked with a secondary antibody (Cell Signaling Technology, Inc.) diluted to 1:100 in PBS for 1 h at room temperature. Immunofluorescence was observed using a microscope (DM6000B; Leica, Wetzlar, Germany).

### Tumor Xenograft Assay

Due to the stronger tumorigenic ability of A549 cells compared to H1299 cells, A549 cells are more frequently employed in subcutaneous tumor formation assay. we conducted subcutaneous tumor transplantation assay in nude mice using A549 cells.

A549 cells (2×10^6^) transfected with NEDD1 were subcutaneously injected into the back of BALB/c female nude mice (6-8 weeks) (HFK Bio-Technology, Beijing, China), as previously described. The tumor growth curve was plotted to observe the effects of NEDD1 on tumor formation and growth *in vivo*. Tumor volume was measured every 5 days for 1 month. The volume was calculated based on the formula: Π/6×length×width. After the 30th day, the mice were sacrificed, the tumor masses were collected, and the volume and weight were measured.

### Tissue Samples

Samples of carcinomas and adjacent normal tissues were obtained from surgical specimens from patients with lung cancer at Yantaishan Hospital and frozen in liquid nitrogen immediately after surgical removal and maintained at -80 °C for protein ex-traction. All studies were approved by the Ethics Committee of Binzhou Medical University, and informed consent was obtained from all the patients.

### Clinical Data Collection and Immunohistochemistry

Lung cancer data and clinical information of all patients whose samples were included in the tissue microarray were collected from Shanghai; detailed information is presented in Table 1.

Immunohistochemistry was performed according to routine operations. The sections were subjected to deparaffinization and antigen retrieval with 10 mM sodium citrate (pH 6.0) solution, and blocked with PBS containing 5% goat serum for 30 min RT. The primary antibody was diluted in PBS containing 5% goat serum (1:200), added to the slides and incubated overnight at 4 °C, followed by incubation with the HRP-conjugated secondary antibody and staining with the 3.30-diaminobenzidine (DAB) substrate.

For quantitative analysis, the tissue score (IHC score) was calculated based on tissue staining intensity and percentage of stained cells. The intensity scoring standards were as follows: 0, cells not stained; 1, weakly stained compared to stromal cells; 2, moderately stained; 3, strongly stained; and 4, more intensely stained. The percentage of stained cells was calculated as 0-100%, and the H score was calculated by multiplying the intensity score by the percentage score to the range between 0 and 400. Tissue scoring was performed by two independent researchers blinded to the clinicopathological results. These features were included in further analysis.

### Comet Experiment

Comet assay (single-cell gel electrophoresis assay) was performed according to the manufacturer's protocol (Trevigen, America). Cells were harvested and suspended with low-melting-point agarose (1%), layered onto adhesive microscope slides pre-coated with 0.5% normal melting point agarose. Later, the slides were submerged in lysis buffer for two hours at 4 °C, alkaline electrophoresis (pH>13) was performed for 30 minutes; subsequently, the slides were neutralized with 0.4mM Tris-HCL (pH 7.5) buffer and stained with PI. The analysis was performed with fluorescence microscopy (DM6000B; Leica, Wetzlar, Germany). The DNA damage was analyzed using Comet Assay Analysis Software (CASP1.2.3b2). Based on the percentage of comet tail fluorescence intensity accounting for the total intensity (Tail DNA%), the captured cellular images are categorized into 5 grades. grade 0<5%, grade1: 5%~20%, grade2: 20%~40%, grade3: 40%~95%, grade4: >95%.

### Statistical Analysis

In cellular and animal experiments, data were collected from three isolated experiments and presented as mean ± SEM and analyzed by GraphPad Prism 5.0 (GraphPad Software, San Diego, CA, USA). Statistical significance was assessed using the student's t-test and two-way analysis of variance. Statistical significance was set at *p* < 0.05.

To analyze the relationship between clinicopathological characteristics and NEDD1 expression at mRNA and protein levels, mRNA expression of the lung cancer datasets in The Cancer Genome Atlas (TCGA) which were downloaded from the UCSC Xena (https://xena.ucsc.edu/ (accessed on 1 February 2021)) and the NEDD1 immune score of one tissue microarray system containing 98 cases were included in the study. All statistical analyses were performed with R 4.0.0 software (R Foundation for Statistical Computing, Vienna, Austria, packages “pheatmap,” “ggplot2,” “tableone,” “survival,” “survminer”) through R Studio 1.2.1335 software environment (PBS, Boston, MA, USA) and IBM SPSS 26.0 (IBM, Chicago, IL, USA). The clinicopathological features of LUAD patients and NEDD1 immune score were described using summary statistics, with continuous variables shown as median (IQR) and categorical variables as frequencies (N) and percentages (%). The Mann-Whitney U test or Kruskal-Wallis H test was used to determine the significance of differences between the risk score and clinicopathological characteristics. The Wilcoxon rank sum test was performed to determine significant differences in paired data. The chi-square (*χ^2^*) test was used to analyze the correlation between NEDD1 immune score level and clinicopathological parameters. The contingency coefficient was calculated to confirm the independence of clinicopathological parameters. Kaplan--Meier survival curves and log-rank tests were used to analyze survival differences between the high-score (≥ median of NEDD1 immune score) and low-score (< median of score) groups. Univariate and multivariate Cox proportional hazard models were used to estimate the hazard ratios of the prognostic factors. All statistical tests were two-sided; *p* < 0.05 was considered statistically significant.

## Results

### NEDD1 is Overexpressed in LUAD and Contributes to a Poor Prognosis

**Figure 1:**
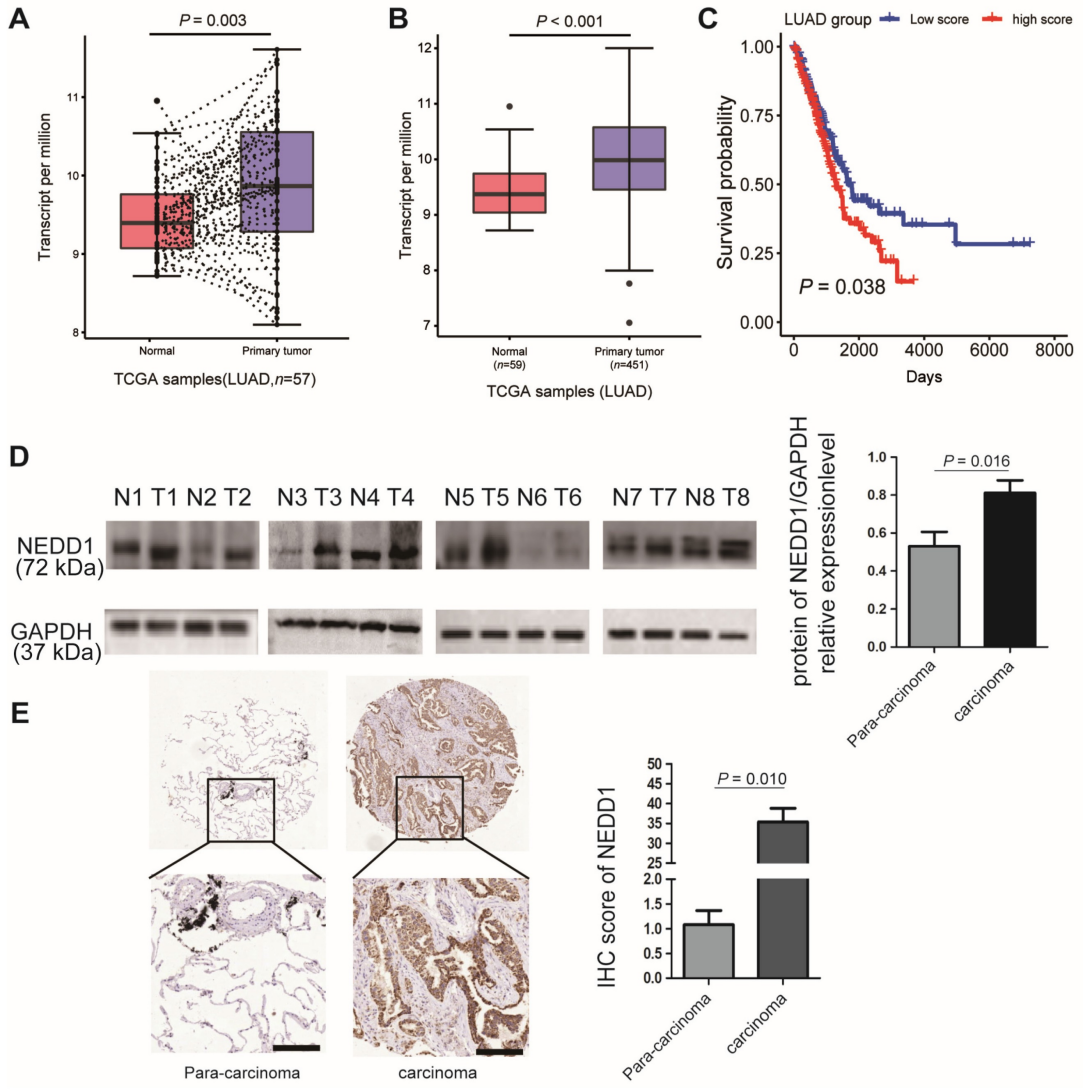
NEDD1 expression and survival difference analysis of NSCLC. **(A)** Expression difference between primary tumor tissues and paired normal tissues of lung adenocarcinoma (LUAD) patients in TCGA dataset were analyzed by paired t-test. **(B)** Sensitivity analysis was adopted to assess the stability of expression difference between tumor and normal tissues of LUAD patients in TCGA dataset. 59 normal tissues and 451 tumor tissues were analyzed by student t-test. **(C)** Survival was estimated by the Kaplan-Meier method, and survival difference between patients with high and low risk scores was compared by log-rank test. **(D)** Expression difference between normal tissues and tumor tissues of eight patients with LUAD were detected by Western blot and Image J was used to analysis data. **(E)** Expression difference between normal tissues and tumor tissues of 98 patients with LUAD were detected using immunohistochemistry bar=200 μm, and the IHC staining score was calculated with Image-Pro Plus , the statistical analysis was performed using the student's t-test and two-way analysis of variance.

Because gene dysfunction is always accompanied by abnormal expression, it is vital to detect the expression level of NEDD1 in clinical lung carcinoma samples. The most prevailing types of lung cancer are adenocarcinoma and squamous cell carcinoma; when analyzing the TCGA database, we found that the mRNA expression of NEDD1 in LUAD tissues was much higher than that in regular lung tissue from the same individual (Fig. 1A). The sensitivity analysis also revealed that NEDD1 was up-regulated in LUAD tissues compared with in normal lung tissues (*p* < 0.001, Fig. 1B). Moreover, as shown by Kaplan-Meier survival curves, lung cancer patients with high NEDD1 expression had significantly poor prognoses than those with low NEDD1 expression (Fig. 1C).

To validate the results of bioinformatics analysis, the relative NEDD1 expression in LUAD tissues and adjacent non-tumor tissues from eight cases were detected by western blot and analyzed using ImageJ (*p* = 0.016, Fig. 1D); the results confirmed that NEDD1 was up-regulated in LUAD tissues compared to non-tumor tissues. To identify NEDD1 overexpression at the protein level, we used immunohistochemistry to detect NEDD1 in a LUAD tissue array comprising 98 cases and found a similar significant difference between adenocarcinoma tissues and normal lung tissues (*p* = 0.010, Fig. 1E).

### Association between NEDD1 Expression and Clinicopathological Characteristics of Patients with LUAD

**Figure 2:**
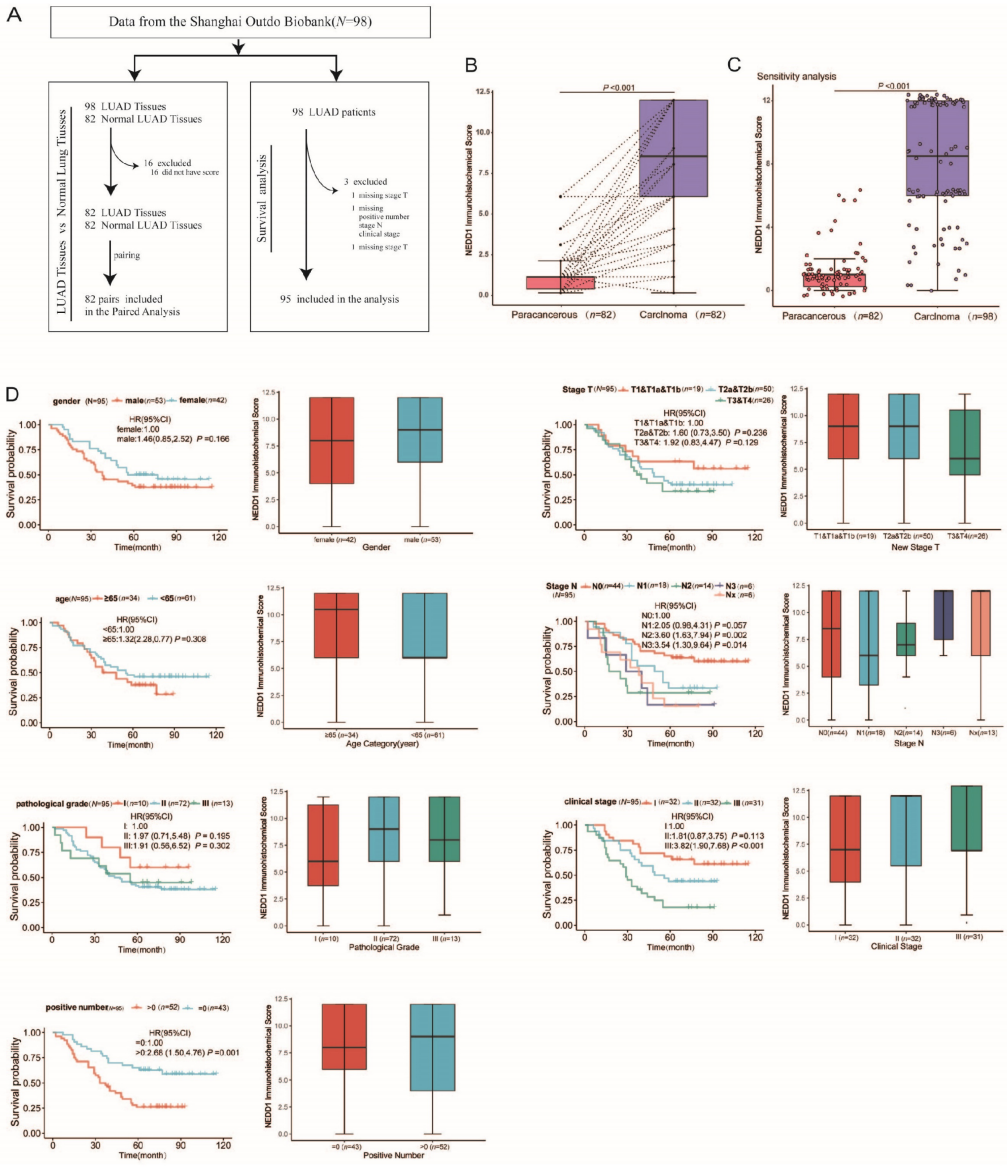
Association between NEDD1 Expression and Clinicopathological Characteristics of Patients with LUAD. **(A)** Flow chart for the LUAD patient selection from the Shanghai Outdo Biobank. **(B)** Paired differentiation analysis for the immune score of NEDD1 in LUAD samples and the paired normal samples de-riving from the same patients (*p* < 0.001 by Wilcoxon test). **(C)** Sensitivity analysis: Differentiation analysis of the immune score of NEDD1 in total LUAD samples and normal samples (*p* < 0.001 by Mann-Whitney U test). **(D)** Association of NEDD1 immune score with survival and clinicopathological features. The boxplots indicate the association of NEDD1 immune score with different clinicopathological features by the Mann-Whitney U test or Kruskal-Wallis H test. The Kaplan-Meier curves of overall survival for LUAD patients was plotted. The log-rank test was carried out for survival analysis.

From July 2004 to June 2009, a total of 98 LUAD patients underwent surgery and were followed up for ten years until August 2014. Finally, 95 patients (three samples without clinicopathological characteristics) with LUAD having complete data were enrolled (Fig. 2A). The median survival after surgency was 49 months, with 42 LUAD patients (42.9%) still alive at the end of the follow-up period. The patients' age ranged from 20-81 years, with a median of 60 years. Regarding the pathological grade, 10.2% were grade I, 75.5% were grade II, and 14.3% were grade III. The total number of lymph nodes ranged from 1-38, with a median of 9. The main clinicopathological characteristics of all patients with LUAD are summarized in Table 1.

In order to explore the underlying associations between the NEDD1 immune score and lung tissue type, we analyzed the lung tissue type of patients with LUAD with respect to the NEDD1 immune score. Notably, there were significant differences in the NEDD1 immune score in LUAD tissues compared with regular tissues (*p* < 0.001, Fig. 2B). The sensitivity analysis also revealed that NEDD1 was up-regulated in LUAD tissues compared to paired normal lung tissues from the same patients (*p* < 0.001, Fig. 2C).

Clinicopathological features, including sex, age, pathological grade, positive number, pathological T stage, pathological N stage, and clinical stage, were collected from the Shanghai Outdo Biobank. The results of the rank sum test showed that seven types of clinicopathological features were not significantly correlated with the NEDD1 immune score. However, Kaplan-Meier survival analysis showed that the positive number, pathological N stage, and clinical stage were significantly correlated with survival of patients with LUAD (Fig. 2D).

### High NEDD1 Expression is Related to Worse Survival in Patients with LUAD

**Figure 3:**
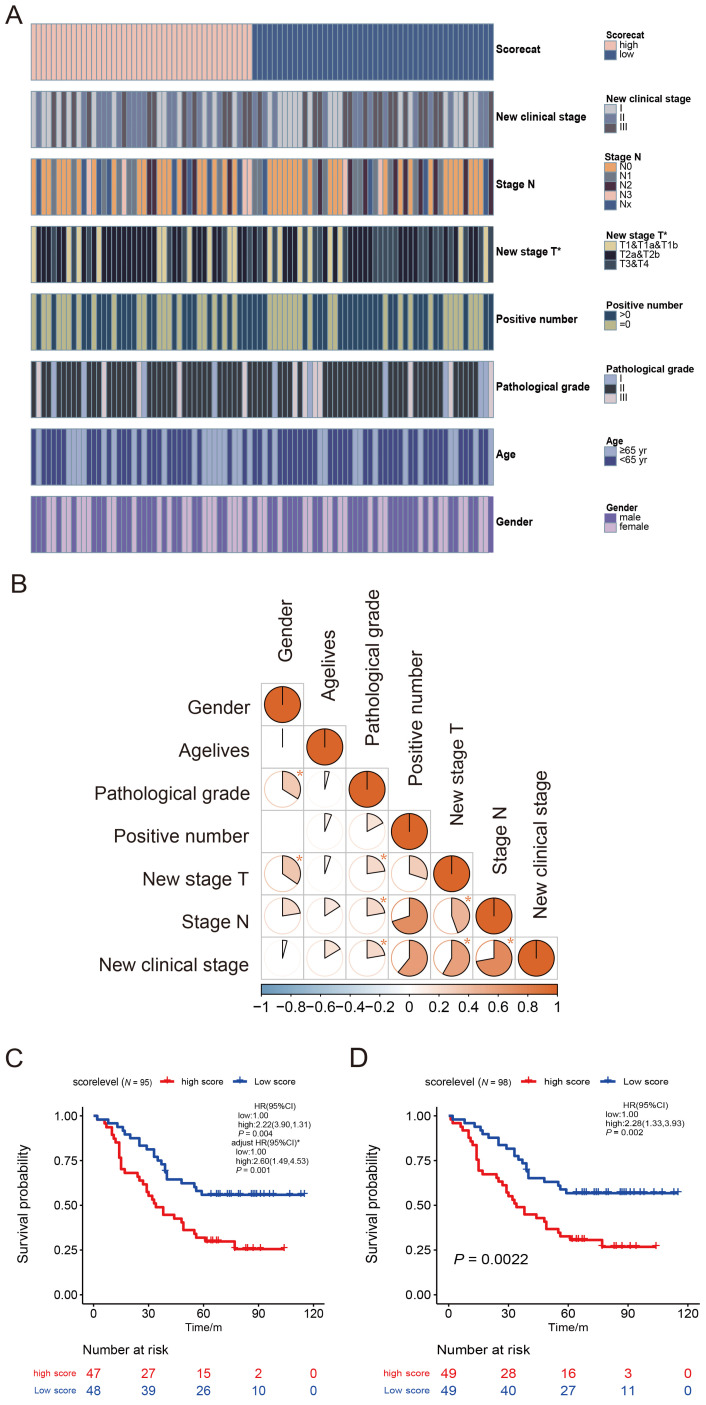
Survival analysis of overall survival for the high immune score and low score groups. **(A)** Heatmap for clinicopathological features conducted by comparing the high immune score group with low score group. Row name: clinicopathological feature name. Column name: IDs of LUAD patients that are not shown in plot. Chi-square test for contingency tables was carried out as the significance test (Supplementary Table 1). * *p* < 0.05. The association between pathological T stage and NEDD1 immune score classes was statistically significant (*χ^2^* = 7.282, *p* = 0.026). **(B)** Heatmap displays the association between seven kinds of clinicopathological features. Each spot represents the correlation value between two kinds of cells. The contingency coefficient was determined for significant tests. **(C)** Kaplan-Meier curves of overall survival for LUAD patients with complete clinical information (N = 95). Crude HR (95% CI): 2.22 (1.31-3.90) measured by the univariate Cox proportional hazards analysis. The adjusted HR (95% CI): 2.60 (1.49-4.53) was measured by the multivariate Cox proportional hazards analysis. The HR was adjusted by 7th AJCC clinical stage. **(D)** Sensitivity analysis: Kaplan-Meier curves of overall survival for total LUAD patients (N = 98). Crude HR (95% CI): 2.28 (1.33-3.93) using the univariate Cox regression model.

Patients with LUAD (N = 95) were divided into high-and low-score groups according to the median cut-off of the NEDD1 immune score. A heat map of NEDD1 immune score classes and clinicopathological features was constructed. Results of the Chi-square test showed that the pathological T stage was significantly correlated with NEDD1 immune score classes (Fig. 3A, Table S1). To further confirm the connection between clinicopathological features, we applied a contingency coefficient (Fig. 3B, Table S2). A high correlation between pathological N stage and positive number (contingency coefficient = 0.700), clinical stage by positive number (contingency coefficient = 0.608), and clinical stage by pathological T stage (contingency coefficient = 0.582) was revealed.

Based on NEDD1 immune score classes and clinical information, we analyzed the survival curves for patients with LUAD by comparing the high-score group (NEDD1 immune score < 8) with the low-score group (≥ 8). There was a significant difference in scores of 95 patients with LUAD (three cases lacked clinical information) between the two groups (*p* = 0.004, Fig. 3C). The Kaplan-Meier survival analysis of all 98 patients with LUAD also showed similar results between groups (*p* = 0.002, Fig. 3D).

To further determine the effect of NEDD1 immune score on Overall Survival, we applied a Cox proportional hazards model. Univariable Cox regression analysis of OS showed that a high NEDD1 immune score was a distinct risk factor for poor survival (HR [95% CI]: 2.22 [1.31-3.90], *p* = 0.004). Some clinicopathological features, including positive number, clinical stage, and pathological N stage, also showed similar results. These results were consistent with those from the Kaplan-Meier curve analysis. Subsequently, the NEDD1 immune score classes, positive number, pathological N stage, and clinical stage were analyzed using multivariable Cox regression analysis. Because of the high correlation between positive rate, pathological T stage, pathological N stage, clinical stage, they were considered entirely in the multivariable Cox regression analysis. After adjusting for the clinical stage in the multivariable analysis, NEDD1 immune score classes remained an independent prognostic factor of OS (adjusted HR [95% CI]: 2.60 [1.49-4.53], *p* = 0.001, Fig. 3C). The detailed results of the Cox regression survival analysis of OS are shown in Table 2.

### NEDD1 Promotes Proliferation and Metastasis of LUAD Cells

**Figure 4:**
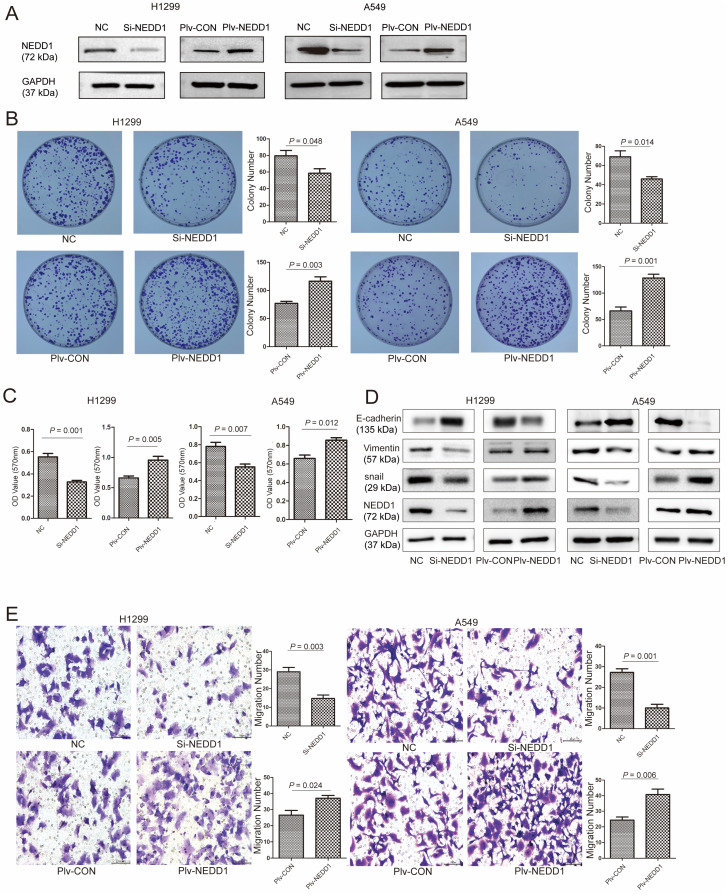
NEDD1 promotes proliferation and metastasis of lung adenocarcinoma cells. **(A)** NEDD1 was knockdown/overexpressed in H1299 and A549 cells respectively. **(B)** Colony formation assay in H1299 and A549 cells treated as indicated and the related analysis. Data are ex-pressed as the mean ± SEM of triplicate experiments. **(C)** MTT assay showing H1299/A549 cells after transfection with si-NEDD1 or overexpression. Data were expressed as the mean ± SD from triplicate experiments; **(D)** Western blot analysis the expression of EMT-related proteins; GAPDH was used as a control in H1299/A549 cells. **(E)** Cell migration ability was investigated by trans-well migration assays in H1299 and A549 cells after NEDD1 was either inhibited or overexpressed. Statistical analysis of the migrated cells is shown (bar =100 µm). Data are expressed as the mean ± SD of triplicate experiments; Student's t-test

NEDD1 was knockdown or overexpressed in H1299 and A549 cells, respectively (Fig. 4A). To determine the biological roles of NEDD1 in lung carcinogenesis, we detected the proliferation and metastasis of lung cancer cells using gain-of-function or loss-of-function assays.

In colony formation assays, NEDD1 up-regulation notably increased the number of colonies of H1299 cells (*p* = 0.048) and A549 cells (*p* = 0.014) compared to the control groups. In contrast, NEDD1 knockdown in H1299/A549 cells markedly inhibited cell clonogenicity (Fig. 4B). Consistently, in the 3-(4,5-Dimethylthiazol-2-yl)-2, 5-diphenyl-2H-tetrazol-3-ium bromide (MTT) assay, compared with the control groups, there was an apparent decrease in cell viability in both H1299 cells (*p* = 0.001) and A549 cells (*p* = 0.007), when NEDD1 expression was knocked down. In contrast, NEDD1 overexpression facilitated the proliferation of H1299 and A549 cells (Fig. 4C).

Epithelial-mesenchymal transition (EMT) is associated with tumor initiation, invasion, metastasis 18, and drug resistance 19, 20. To identify the relationship of EMT with NEDD1, we detected the expression of EMT related proteins in H1299/A549 cells using western blot and found that knocking down NEDD1 increased E-cadherin expression and decreased the expression of vimentin and snail. In contrast, NEDD1 overexpression caused the opposite effects by down-regulating E-cadherin, but up-regulating vimentin and snail expression (Fig. 4D).

In addition, the transwell assay showed that the number of migrated cells treated with si-NEDD1 was lower than that in the control group, but that introducing NEDD1 enhanced cell migration (Fig. 4E).

### NEDD1 Regulates Apoptosis and Cell Cycle Progression

**Figure 5:**
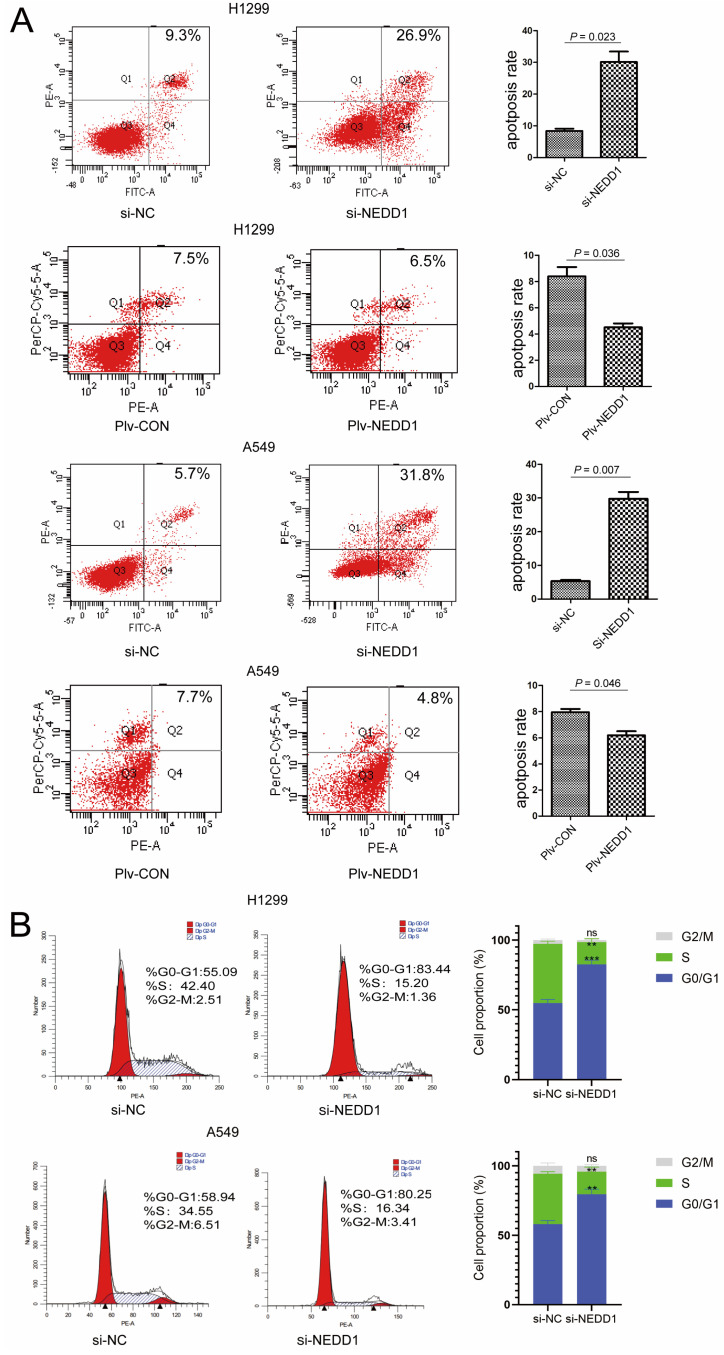
NEDD1 regulates apoptosis and cell cycle progression. **(A)** Apoptotic rate of H1299 and A549 cells with indicated treatment were measured via flow cytometry from triplicate experiments and the related analysis. **(B)** Flow cytometry analysis for cell cycle distribution in H1299 and A549 cells after knocking down NEDD1(**P<0.01, ***P<0.001).

Since dysfunction of chromosome formation results in mitosis failure and apoptosis, to identify the mechanism by which NEDD1 affects the tumorigenesis of LUAD, we examined the effects of NEDD1 on apoptosis and cell cycle progression. First, flow cytometry was used to analyze apoptosis after NEDD1 overexpression or down-regulation in H1299 and A549 cells. We found that NEDD1 down-regulation can significantly increase the apoptosis rate of both cell lines. Consistently, the apoptosis rate of cells overexpressing NEDD1 was reduced compared to that in the control group (Fig. 5A).

We analyzed the effect of NEDD1 on the cell cycle progression of A549 (p53 wild-type) and H1299 (p53-deficient) cells. We detected the distribution of cell cycle status using Flow cytometric analysis (FCM) after PI staining, and found that the percentage of G1 phase cells was significantly increased in NEDD1 silencing A549 cells compared to that in control cells. This is like the results in A549 cells; H1299 cells treated with siNEDD1 were also arrested in the G1/S-phase, while the number of cells in the S-phase was significantly decreased. These results indicate that NEDD1 knockdown resulted in apoptosis and G1-S cell cycle transition arrest (Fig. 5B).

### NEDD1 Deprivation Leads to DNA Damage

**Figure 6:**
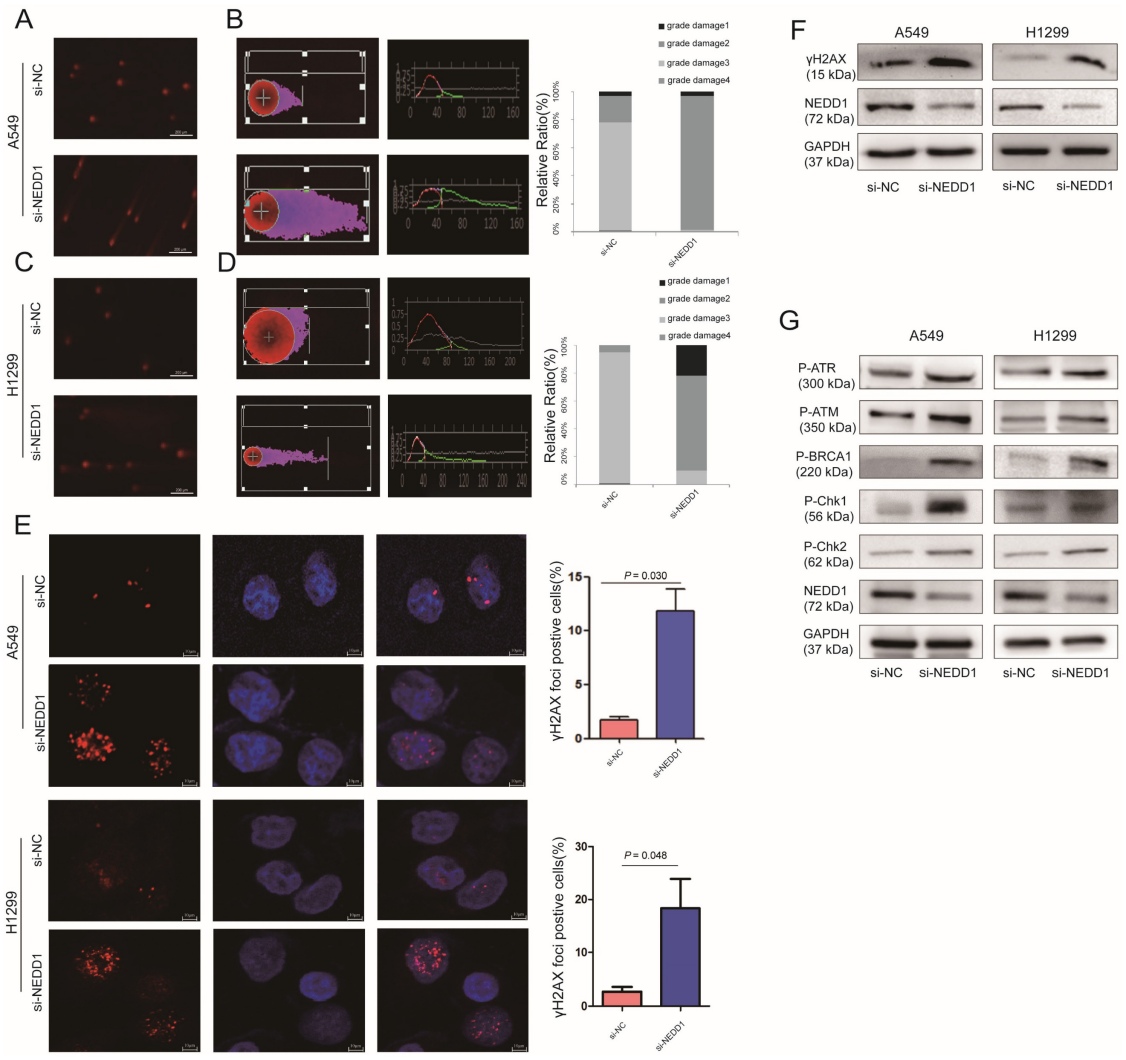
Down regulation of NEDD1 results in DNA damage. **(A)**The comet assay in NEDD1-deficient A549 cells.** (B)** DNA damage was evaluated with tailing situations in A549 cells. Image of tailing situations in H1299 cells(left) and statistical analysis(right) are shown. **(C)**The comet assay in NEDD1-deficient H1299 cells. **(D)** DNA damage was evaluated with tailing situations in H1299 cells. Image of tailing situations in H1299 cells(left) and statistical analysis(right) are shown. **(E)** γH2AX foci in A549 cells and H1299 cells treated with siRNAs were detected by immunostaining (red), and the nuclei were stained with DAPI (blue), for each group, the focal points of 60 cells randomly selected were counted and analyzed. **(F)** The expression of γH2AX and NEDD1 were measured by western blot in A549 cells and H1299 cells under knockdown of NEDD1 with siRNAs. **(G)** Western blot detection of DNA damage and repair-related proteins in A549 cells and H1299 cells under knockdown of NEDD1 with siRNAs.

Given that NEDD1 is crucial for meiotic spindle stability and accurate chromosome segregation for cancer therapy, inhibitors or chemotherapy medicine targeting tubulin and mitosis always lead to DNA damage 21. To evaluate the effect of NEDD1 depletion on chromosome stability and the feasibility of NEDD1 as a therapeutic target for LUAD, DNA damage was evaluated using a comet assay following NEDD1 deprivation in A549 cells (Fig. 6A).

Tail DNA (%) and tail length (distance of DNA migration from the nucleus, μm; TL) were also manually scored. Statistical analysis shows that compared with the control group, after the targeted knockdown of NEDD1, DNA damage is more serious. DNA damage in the control group was mainly concentrated in grades 1 and 2, with NEDD1 deprivation, the degree of DNA damage was significantly increased compared to that in the controls, and even grades 2 and 3 damage increased to 100% (Fig. 6B). In H1299 cells, DNA damage was also measured using comet assay (Fig. 6C). Statistical analysis shows that DNA damage is more serious in NEDD1-depleted H1299 cells compared with that in control cells. The grade 3 damage was accounted for 25% of the total (Fig. 6D).

γH2AX foci was considered the marker of DNA damage 22, 23, radiotherapy and cancer drugs used treatments induce DNA double-strands break and the formation of γH2AX 24-26. To further confirm the effect of NEDD1 deprivation on DNA breaks, immunofluorescence staining was performed using antibodies against γH2AX. A549 cells and H1299 cells treated with si-NEDD1 showed significantly more double-strand damage foci (*p* = 0.030, *p* = 0.048, Fig. 6E) and higher γH2AX expression (Fig. 6F). Since DNA damage usually activates the damage repair system, we detected the expression of proteins involved in DNA damage repair by western blot and found that NEDD1 knockdown in both H1299 and A549 cells induced higher P-ATR, P-ATM, P-BRCA1, P-Chk1, and P-Chk2 expression (Fig. 6G).

### NEDD1 Promotes Tumor Growth *in Vivo*

**Figure 7:**
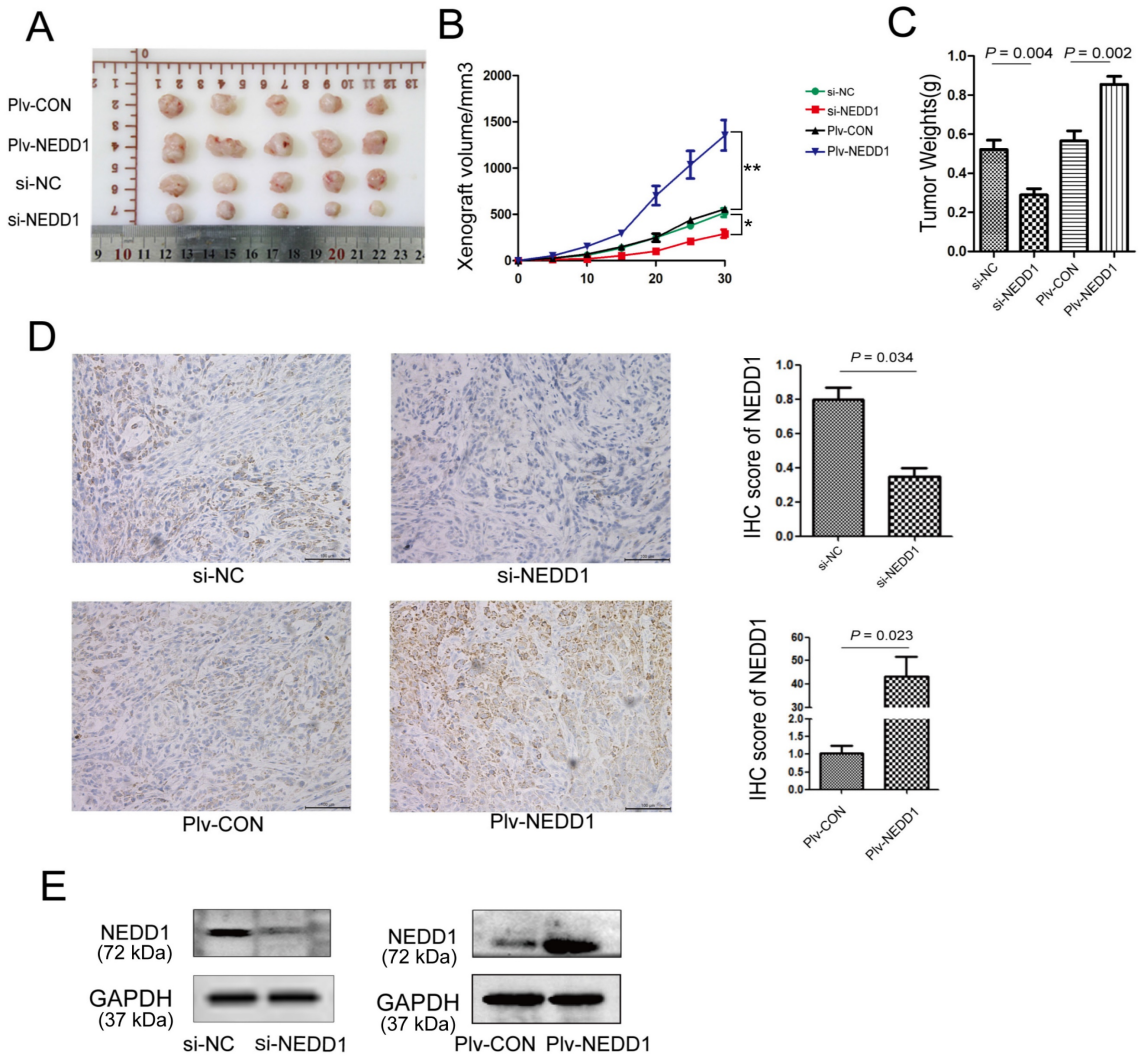
NEDD1 promotes tumor growth *in vivo*. **(A)** The transplanted tumors of nude mice were dissected at the end of experiment. **(B)** The volumes of xenografts were monitored over time (*P<0.05, **P<0.01). **(C)** Tumor weight analysis was performed after tumor dissection; data were expressed as median (interquartile range). **(D)** Expression of NEDD1 in transplanted tumors was detected with IHC (bar=100 μm). **(E)** Western blot analysis of NEDD1 expression in transplanted tumor tissues. GAPDH was used as the inner control.

To further establish the role of NEDD1 in LUAD progression, mouse subcutaneous xenograft transplantation models were established by injecting A549 cells transfected with siRNAs or with a lentivirus (Fig. 7A). Subcutaneous growth was monitored and measured at days 5, 10, 15, 20, 25, and 30. Results of tumor volume (Fig. 7B) and tumor weight (Fig. 7C) analysis shown that tumor growth in NEDD1-overexpressing group was promoted (*p* = 0.002); on the contrary, the growth of tumor *in vivo* was significantly suppressed in NEDD1- deficient groups (*p* = 0.004).

To determine the expression of NEDD1 in tumor xenografts, western blot and immunohistochemistry were performed. We found that NEDD1 was up-regulated in the NEDD1 group and down-regulated in the si-NEDD1 transfected A549 cells group than in corresponding control groups (Fig. 7D and 7E).

## Discussion

A total of 95 patients were included in the survival analysis. The NEDD1 immune high-score group accounted for 49.4% of the patients and the low-score group for 50.6%. The Kaplan-Meier plots showed that patients with high NEDD1 immune scores had worse OS (*p* = 0.003). Multivariable Cox regression analysis also showed that the NEDD1 immune score was an independent prognostic factor.

The finding of this study suggests that NEDD1 was up-regulated in LUAD tissues at mRNA and protein levels. Moreover, patients with low NEDD1 expression had a good prognosis, while those with high expression had a poor prognosis. Research about NEDD1 mainly focuses on the roles of NEDD1 in centrosome formation and microtubule nucleation. Our results reveal that NEDD1 participates in LUAD development as a promoter and support that NEDD1 is a potential prognosis marker for LUAD. However, the mechanism leading to the up-regulation of NEDD1 is unclear. We believe that revealing the cause of NEDD1 overexpression in tumor tissue is of great significance for understanding tumor occurrence.

The NEDD1 or γ-tubulin interaction is critical for spindle assembly and microtubule nucleation. Recently, one study found that NEDD1 phosphorylation by PLK4 facilitates the initiation of the cartwheel assembly and daughter centriole biogenesis 27. Our results indicated a significant NEDD1 overexpression in non-small cell lung cancer (NSCLC), which might promote cartwheel assembly and daughter centriole biogenesis initiation and promote cell proliferation; Centrosome formation and microtubule nucleation are important targets of chemotherapy 21, 28. Increasing evidence suggests dysfunction of centrosome microtubule nucleation in cancer cells. Some proteins related to microtubule nucleation have been regarded as target molecules for tumor therapy. For instance, the kinesin Eg5, which interacts with NEDD1 29, has a similar subcellular location as NEDD1 and is overexpressed in several solid tumors, including lung cancer 30, 31, which leads to genomic instability and promotes cancer progression. Several Eg5 inhibitors have entered clinical trials and demonstrated clinical efficacy in patients with multiple myeloma cancer 32, 33. We found that NEDD1 knockdown inhibits lung cancer cells proliferation, migration, EMT, and tumor formation *in vivo*. Therefore, these factors and NEDD1 depletion also induce DNA damage, indicating that NEDD1 is a putative therapeutic target.

In this report, we analyzed the effects of NEDD1 on cell cycle progression using FCM and found that NEDD1 knockdown blocked the cell cycle in the G1/S phase. The results were similar in A549 cells and H1299 cells. However, this is not consistent with previous reports where it was considered that NEDD1 regulates the cell cycle in a p53-dependent manner, suggesting the absence of functional p53 cells arrest in mitosis, while in cells expressing wild type p53, the cycle already stops in G1/S 34. Although we repeatedly verified the cell cycle assay, we did not observe that the p53-negative cells H1299 cells were blocked in mitosis. Further, in apoptosis assays, silencing of NEDD1 led to >30% apoptosis in both A549 and H1299 cells. In this study, we also observed severe DNA damage after NEDD1 knockdown, and the activation of DNA damage repair system, these events can also lead to cell apoptosis and cell cycle arrest. Therefore, we believe that NEDD1 deletion leads to cell division disorder, DNA breakage, followed by apoptosis and cell cycle arrest.

Proteins with the same domain usually share many similar characteristics. Like NEDD1, WDR5 also contains the WD40 repeat domain, can inhibit p53 ubiquitination, and up-regulate it to influence proliferation and apoptosis of lung cancer cells 35. NEDD4 and NEDD8, of the same family as NEDD1, are closely related to the degradation pathway of protein ubiquitination 36-38. We previously reported that NEDD1 interference in A549 cells up-regulated the p53 expression 39, but we did not explore the regulatory mechanism. Another study reported that NEDD1 and p300 exist in the same complex regulating p53 expression 40; however, whether NEDD1 can regulate p53 ubiquitination, thereby regulating cell proliferation and apoptosis, remains unclear and warrants further investigation. On the other hand, NEDD1 may also interact with other proteins through the WD40 domain and participate in other unknown processes.

A limitation of our study is its retrospective nature, despite trying to include as many clinical features as possible for more rigorous validation of our biomarker.

In summary, our results suggest that NEDD1 plays a vital role in the development of LUAD and may be a potential prognostic marker and promising therapeutic target for lung cancer therapy.

## Supplementary Material

Supplementary tables.

## Figures and Tables

**Table 1:** The patients' clinicopathological characteristics (N=98).

Characteristic	Alive (N=42)	Dead (N=56)	Total (N=98)
Gender - no. (%)			
Male	21 (50.0)	34 (60.7)	55 (56.1)
Female	21 (50.0)	22 (39.3)	43 (43.9)
Age			
Median [IQR]	59.0 [53.0, 65.0]	61.0 [54.0, 72.0]	60.0 [53.25, 66.0]
Category - no. (%)			
< 65 yr	30 (71.4)	33 (58.9)	63 (64.3)
≥ 65 yr	12 (28.6)	23 (41.1)	35 (35.7)
Pathological grade - no (%)			
I	6 (14.3)	4 (7.1)	10 (10.2)
II	29 (69.0)	45 (80.4)	74 (75.5)
III	7 (16.7)	7 (12.5)	14 (14.3)
Total number of lymph nodes			
Median [IQR]	8.0 [5.0, 13.75]	9.0 [6.0, 12.50]	9.0 [5.0, 13.0]
Positive number			
Median [IQR]	0.0 [0.0, 1.0]	2.0 [0.0, 6.0]	1.0 [0.0, 4.0]
Category - no. (%)			
= 0	26 (61.9)	17 (30.4)	43 (43.9)
> 0	16 (38.1)	38 (67.9)	54 (55.1)
Unknown	0	1 (1.8)	1 (1.0)
Stage T - no. (%)			
T1	1 (2.4)	0	1 (1.0)
T1a	1 (2.4)	3 (5.4)	4 (4.1)
T1b	9 (21.4)	6 (10.7)	15 (15.3)
T2a	17 (40.5)	23 (41.1)	40 (40.8)
T2b	3 (7.1)	7 (12.5)	10 (10.2)
T3	7 (16.7)	14 (25.0)	21 (21.4)
T4	2 (4.8)	3 (5.4)	5 (5.1)
Unknown	2 (4.8)	0	2 (2.0)
Stage N - no. (%)			
N0	27 (64.3)	17 (30.4)	44 (44.9)
N1	6 (14.3)	12 (21.4)	18 (18.4)
N2	4 (9.5)	10 (17.9)	14 (14.3)
N3	1 (2.4)	5 (8.9)	6 (6.1)
Nx	4 (9.5)	11 (19.6)	15 (15.3)
Unknown	0	1 (1.8)	1 (1.0)
Stage M - no. (%)			
M0	42 (100.0)	55 (98.2)	97 (99.0)
M1b	0	1 (1.8)	1 (1.0)
Clinical stage - no. (%)			
Stage IA	9 (21.4)	5 (8.9)	14 (14.3)
Stage IB	11 (26.2)	7 (12.5)	18 (18.4)
Stage IIA	8 (19.0)	6 (10.7)	14 (14.3)
Stage IIB	4 (9.5)	2 (3.6)	6 (6.1)
Stage II	4 (9.5)	10 (17.9)	14 (14.3)
Stage IIIA	3 (7.1)	16 (28.6)	19 (19.4)
Stage IIIB	3 (7.1)	7 (12.5)	10 (10.2)
Stage III	0	1 (1.8)	1 (1.0)
Stage IV	0	1 (1.8)	1 (1.0)
Unknown	0	1 (1.8)	1 (1.0)
NEDD1 Immunohistochemical Score			
Median [IQR]	6.0 [4.0, 9.0]	12.0 [6.0, 12.0]	8.50 [6.0, 12.0]
Category - no. (%)			
Low score (< 8.5)	28 (66.7)	21 (37.5)	49 (50.0)
High score (≥ 8.5)	14 (33.3)	35 (62.5)	49 (50.0)

**Table 2:** Univariable and Multivariable Analysis of Prognostics of OS in LUAD.

Characteristic (N=95)	Univariate		Multivariate	
	HR (95%CI)	*p* value	HR (95%CI)	*p value*
NEDD1 Immune Score - Category				
Low score	Ref		Ref	
High score	2.22 (1.31, 3.90)	0.004	2.60 (1.49, 4.53)	0.001
Gender				
Female	Ref		-	
Male	1.46 (0.85, 2.52)	0.166		
Pathological Grade				
I	Ref		-	
II	1.97 (0.71, 5.48)	0.195		
III	1.91(0.56, 6.52)	0.302		
Age - Category				
< 65 yr	Ref		-	
≥ 65 yr	1.32 (0.77, 2.28)	0.308		
Positive Number - Category				
=0	Ref		-	
>0	2.68 (1.50, 4.76)	0.001		
Pathological T Stage				
T1&T1a&T1b	Ref		-	
T2a&T2b	1.60 (0.73, 3.50)	0.236		
T3&T4	1.92 (0.83, 4.47)	0.129		
Pathological N Stage				
Stage N0	Ref		-	
Stage N1	2.05 (0.98, 4.31)	0.057		
Stage N2	3.60 (1.63, 7.94)	0.002		
Stage N3	3.54 (1.30, 9.64)	0.014		
Stage Nx	3.49 (1.62, 7.49)	0.001		
Clinical Stage				
Stage IA& IB	Ref		Ref	
Stage IIA& IIB& II	1.81 (0.87, 3.75)	0.113	1.68 (0.81, 3.50)	0.164
Stage IIIA& IIIB& III& IV	3.82 (1.90, 7.68)	<0.001	4.28 (2.11, 8.68)	<0.001

**Note**: Since the factors positive rate, pathological T stage and pathological N stage are highly correlated with clinical stage (Fig.3B), they weren't included simultaneously in the same multivariable cox model to avoid multicollinearity cox model to avoid multicollinearity. The factor, clinical stage, was included in the multivariable cox model due to its clinical significance.
