# Supplementary material for: NEDD1 Promotes the Development of Lung Adenocarcinoma and Can be Used as a Prognostic Marker
Source: J Cancer. 2024 Aug 13;15(16):5149–64. doi: 10.7150/jca.98238 (PMC11375547; doi:10.7150/jca.98238)
Supplement: Supplementary file 1 — Supplementary tables. [file jcav15p5149s1.pdf]

Positive Number – Category Table S1 Chi-square test for LUAD patients according to the NEDD1 immune score level stratified by clinicopathological characteristics

Characteristics	NEDD1 immune score level		χ^2	p value
	low score	high score		
new clinical stage			2.046	0.359
Stage I	17	15		
Stage II	13	19		
Stage III	18	13		
stage N			4.611	0.330
N0	22	22		
N1	11	7		
N2	9	5		
N3	2	4		
Nx	4	9		
gender			0.255	0.614
female	20	22		
male	28	25		
age			3.200	0.074
< 65 yr	35	26		
≥ 65 yr	13	21		
stage T			7.282	0.026
T1&T1a&T1b	8	11		
T2a&T2b	21	29		
T3&T4	19	7		
Age-Category			0.090	0.765
= 0	21	22		
> 0	27	25		
pathological grade			0.522	0.770
I	6	4		
II	35	37		
III	7	6		

Table S2 Contingency coefficient test of clinicopathological characteristics for 95 LUAD patients

Characteristics	gender	Age-Category	pathological_grade	Positive Number – Category	Stage T	Stage N	new_clinical_stage
gender	-						
Age-Category	0.001	-					
pathological_grade	0.339*	0.04	-				
Positive Number – Category	0	0.061	0.171	-			
Stage T	0.347*	0.053	0.226	0.3*	-		
Stage N	0.225	0.159	0.222	0.7*	0.445*	-	
new_clinical_stage	0.041	0.164	0.223	0.608*	0.582*	0.721*	-

note: * $p < 0.05$
